# Effect of *Lactiplantibacillus plantatum* HFY11 on Colitis in Mice

**DOI:** 10.3390/foods13101496

**Published:** 2024-05-12

**Authors:** Fang Tan, Xianrong Zhou, Lixuan Ren, Chang-Suk Kong

**Affiliations:** 1 Department of Bioscience, Silla University, Busan 46958, Republic of Korea; g-202311663@sillain.ac.kr (F.T.); zxr960032412@sillain.ac.kr (X.Z.); g-202311664@sillain.ac.kr (L.R.); 2 Department of Food and Nutrition, Silla University, Busan 46958, Republic of Korea; 3 Marine Biotechnology Center for Pharmaceuticals and Foods, Silla University, Busan 46958, Republic of Korea

**Keywords:** *Lactiplantibacillus plantatum*, colitis, intestinal microecology, expression, cytokine

## Abstract

This study aimed to examine the potential impact of the intervention of *Lactiplantibacillus plantatum* HFY11 (LP-HFY11) on colitis using in vivo animal trials. The impact of LP-HFY11 intervention on colitis was determined by measuring the levels of relevant indicators in the intestine, colon, and blood after oxazolone-induced colitis in BALB/c mice. The results of the trial show that LP-HFY11 improved the colon weight-to-length ratio, reduced the colitis-induced colon length shortening, and reduced colonic abstinence. Furthermore, it decreased the levels of myeloperoxidase, nitric oxide, and malondialdehyde activities while increasing the glutathione content in the colon tissue of colitis-affected animals. LP-HFY11 lowered the interleukin-10 (IL-10) level and increased the IL-2 level in the serum of colitis mice. LP-HFY11 also upregulated the expression of neuronal nitric oxide synthase, endothelial nitric oxide synthase, c-Kit, and stem cell factor (SCF), and downregulated the expression of IL-8, C-X-C chemokine receptor type 2 (CXCR2), and inducible nitric oxide synthase (iNOS) in the colon tissue of mice with colitis. LP-HFY11 decreased the expression of *Firmicutes* in the gut while increasing the expression of *Bacteroidetes*, *Bifidobacteria*, and *Lactobacillus*. This indicates that LP-HFY11 could control physiological alterations in the serum and colon tissue, as well as the expression of gut microorganism.

## 1. Introduction

Rich in nutrients, yak yogurt is a naturally fermented dish often found in the minority areas of the Qinghai–Tibet Plateau [1]. Yak yogurt offers several physiological benefits, including decreasing cholesterol, boosting the immune system, and acting as an antioxidant. The taste and quality of yak yogurt differ greatly from regular fermented milk due to the distinct natural fermentation environment of the Qinghai–Tibet Plateau, the use of yak milk and special fermentation vessels, and the unique fermentation microorganisms of the Tibetan people [2]. The *Lactiplantibacillus plantatum* HFY11 used in this study was a strain of lactic acid bacteria that can be used for food after the isolation and identification of yak yogurt microorganisms in the Hongyuan area of the Qinghai–Tibet Plateau in Sichuan Province, China. 

Colitis is an inflammatory intestinal disease, mainly involving inflammation and damage to the colon (large intestine) mucosa. The inflammation and damage to the mucosa interfere with the normal digestion and absorption of food, resulting in discomfort and malnutrition. At the same time, patients with colitis often have problems such as loss of appetite and indigestion, leading to weight loss [3]. If colitis is not effectively treated or controlled, the inflammation may further expand and lead to serious complications, such as intestinal obstruction, bleeding, and perforation. This increases the health risks and the need for medical intervention [4]. In addition, patients with colitis may often experience abdominal pain, abdominal discomfort, and colic. Colitis has a multifaceted impact on the human body, which is not only limited to digestive system problems, but may also involve malnutrition, weight changes, pain, and discomfort. Therefore, timely diagnosis, active treatment, and disease management are essential for patients with colitis [5]. Oxazolone can induce a T-cell-mediated immune response. The cell-mediated type 2 (Th2) immune response and interleukin-4 (IL-4) and IL-5 production increase significantly, accompanied by weight loss, diarrhea, ulcers, and a decreased number of colorectal epithelial cells [6]. The oxazolone-induced ulcerative colitis model is frequently used to identify the physiological action of medications and functional foods in colitis in humans because both show similar symptoms.

Probiotics have a certain impact and potential benefits on colitis; they can help restore the balance of intestinal microbiota, enhance the integrity of the intestinal mucosal barrier, and regulate the immune system [7]. Probiotics can reduce the inflammatory response and symptoms in patients with colitis by enhancing the composition and function of the intestinal microbiota, inhibiting the growth of pathogenic bacteria, and increasing the number of beneficial bacteria [8]. They may enhance the quality of life of patients and aid in the relief of digestive system issues such as constipation, diarrhea, and stomach discomfort [9,10,11]. In addition, probiotics can also enhance the integrity of the intestinal mucosal barrier and reduce the penetration of harmful substances and bacteria, and thus reduce the degree of inflammation. They can also regulate the immune response, reduce the release of inflammatory factors, and promote the resolution of inflammation, thus helping to control the progression of colitis [12]. 

*Lactobacillus* with good physiological activity can be developed and used as a probiotic. Our group investigated the intestinal physiological activity of lactic acid bacteria found in yak yogurt from the Qinghai–Tibet Plateau. The findings demonstrate that lactic acid bacteria separated from yak yogurt had an antioxidant and constipation-relieving effect. Thus, this study was performed to investigate the impact of an *L. plantarum* HFY11 (LP-HFY11) strain isolated from yak yogurt on oxazolone-induced colitis. The findings of this study might give us a theoretical foundation for the development and use of LP-HFY11 in the future.

## 2. Materials and Methods

### 2.1. Experimental Microbial Species

In Hongyuan County, Aba Tibetan and Qiang Autonomous Prefecture, Sichuan Province, China, naturally fermented yak yogurt yielded *L. plantarum* HFY11, which was isolated and stored at the China General Microbiological Culture Collection Center (CGMCC, collection number: CGMCC No. 16644). The frozen P2-generation *L. plantarum* HFY11 strains were inoculated into MRS medium at a 2% inoculation rate and incubated at 36 °C for 16 h, and then freeze-dried. Then, the strain’s dry powder was mixed with other feed ingredients in proportion to qualitatively produce mouse feed, and the resulting feed was stored in a refrigerator at −20 °C for use. 

### 2.2. Animal Experiment

Hunan Slake Jingda Experimental Animal Co., Ltd. provided 50 specific pathogen-free (SPF) male BALB/c mice (aged 7 weeks, weighing 25–30 g), bearing animal license number SCXK (Xiang) 2019-0004. Ten BALB/c mice were randomly assigned to each of five groups: the LP-HFY11 low-concentration treatment (LP-HFY11L) group, LP-HFY11 high-concentration treatment (LP-HFY11H) group, gatifloxacin group (positive control group), model group, and normal group. Following a week of adaptive feeding, a patch measuring 2 × 2 cm^2^ was shaved from the abdomen of each mouse. Then, 0.2 mL of analytical pure ethanol was used to smear the mice in the normal and model group, whereas 0.2 mL of a 3% oxazolone solution (*w*/*w*) was used to smear the animals in the other groups. The mice were permitted to consume water freely on the fifth day, despite fasting beforehand. Following a 24 h fast, the mice were given 0.1 mL/10 g of chloral hydrate to induce unconsciousness. Following this, a silicone tube with a blunt end was inserted 3.5 cm deep into the anus of the animals. Then, 0.15 mL of a 50% ethanol solution was injected into the normal group of mice, whereas 0.15 mL of a 1% oxazolone solution (mass ratio, 50% ethanol as the solvent) was injected into the other groups of mice. The catheter was removed after 20 s, and the mice were inverted for 30 s [13]. The mice in the gatifloxacin treatment group were allowed to eat and drink 0.1% (*w*/*w*) gatifloxacin. The mice in the LP-HFY11L and LP-HFY11H groups were allowed to eat and drink 0.01% (*w*/*w*, 10^7^ CFU LP-HFY11/g feed) and 0.1% (*w*/*w*, 10^8^ CFU LP-HFY11/g feed) lyophilized LP-HFY11 powder, and fed with the same diets for 7 days. Subsequently, all of the mice were killed by dissecting their necks, and the plasma was extracted. The colon’s weight and length were then measured using the extracted colon tissue. The disease activity index (DAI) was established concurrently using the following formula: DAI = (weight loss score + stool traits score + fecal blood score)/3 (Table 1). The operations conformed to the experimental ethics requirements of the animal ethics committee of the Chongqing Collaborative Innovation Center for Functional Food (license number: 202311003B).

### 2.3. Determination of MPO, NO, GSH, MDA, and SOD Levels in Mouse Colon Tissue

The cleaned mouse colon was filled nine times with normal saline, and ultrasonic crushing was used to homogenize the colon tissue. Next, the myeloperoxidase (MPO), nitric oxide (NO), glutathione (GSH), malonaldehyde (MDA), and superoxide dismutase (SOD) levels in the mouse colon tissue were determined using corresponding kits following the manufacturer’s protocols (Thermo Fisher Scientific, Waltham, MA, USA).

### 2.4. Determination of Inflammation-Related Cytokines in Mouse Serum

The blood was extracted from the orbit and centrifuged at 4 °C for 10 min at 1500 rpm. After separating the upper layer of the serum, the levels of cytokines IL-2 and IL-10 in the mouse serum were measured using enzyme-linked immunosorbent assay following the kit’s instructions (Thermo Fisher Scientific).

### 2.5. Colon Tissue Slice Detection

Once dissected, the colon tissue from the mice was fixed in 10% formalin. The tissue samples were dehydrated for 48 h before being embedded in paraffin, cut, and stained with hematoxylin and eosin (H&E) (BX53, Olympus, Tokyo, Japan) [14].

### 2.6. Determination of Related Expression in Colon Tissue Using qPCR

Furthermore, 100 mg of tissue from the middle segment of the mouse colon was taken. The tissue was washed with normal saline and then with clean normal saline at a ratio of 1:9. Following the homogenization of the tissue, 1.0 mL of the RNAzol reagent was applied to extract mouse tissue RNA, and the extracted RNA concentration was adjusted to 1 μg/μL. After reverse transcription, cDNA was obtained, and the reaction system was set up. SYBR Green Polymerase Chain Reaction (PCR) Master Mix (10 μL), sterile deionized water (7 μL), cDNA (1 μL), and PCR primers (1 μL each of the forward and reverse primers, each with a concentration of 10 μmol/L, Thermo Fisher Scientific) were all included in the system solution. The amplification conditions for the prepared reaction solution were as follows: 95 °C for 60 s; 95 °C for 15 s, 40 cycles; 55 °C for 30 s; 72 °C for 35 s; 95 °C for 30 s; and 55 °C for 35 s (SteponePlus, Thermo Fisher Scientific). The internal reference gene employed in this study was glyceraldehyde-3-phosphate dehydrogenase (GAPDH) (Table 2), and the 2^−ΔΔCt^ technique was used to determine the relative expression of each gene [15].

### 2.7. Determination of Microbial mRNA Expression in the Intestinal Contents of Mice

Furthermore, 1.0 g of mouse intestinal contents was weighed, and the microbial mRNA expression in the mouse intestinal contents was determined.

### 2.8. Statistical Analysis

Following three iterations of the experiment, the findings of three parallel experiments were averaged. SAS9.1 statistical software (SAS Institute Inc., Cary, NC, USA) was used to determine whether the data from each group differed substantially at the *p* < 0.05 level using one-way analysis of variance.

## 3. Results

### 3.1. Effects of LP-HFY11 on Colitis Symptoms

The experimental results showed that the DAI value of the normal-group mice remained at 0.00 ± 0.00 throughout the experimental cycle (Table 3). On the contrary, the DAI value of the model-group mice increased steadily following oxazolone enema administration and remained the highest of all the groups. However, after administering gatifloxacin and LP-HFY11 via gavage, the DAI value of the oxazolone-induced colitis mice showed a downward trend (*p* < 0.05). Specifically, the DAI value declined more noticeably in the gatifloxacin and LP-HFY11H groups, but the LP-HFY11L group had a lower DAI value than the two treatment groups mentioned earlier.

Based on the results, the model group exhibited the lowest colon length and colon weight-to-length ratio, whereas the normal group showed the highest values for these measurements. The colon length and colon weight-to-length ratio in the LP-HFY11H group were nearly identical to those in the normal group (Table 4), and the outcome was comparable to that of gatifloxacin.

### 3.2. Effects of LP-HFY11 on the Levels of MPO, NO, GSH, and MDA in Mouse Colon Tissue

The colon tissue in the model group (colitis) mice had the lowest content of GSH and the highest levels of MPO, NO, and MDA, as shown in Table 5. However, the colon tissue in the normal group had the highest concentration of GSH and the lowest levels of MPO, NO, and MDA. In the colon tissue of mice with colitis, gatifloxacin, LP-HFY11L, and LP-HFY11H all decreased the levels of MPO, NO, and MDA, while the content of GSH increased compared with those in the model group. Specifically, the LP-HFY11H had an impact equivalent to that of gatifloxacin, bringing the MPO, NO, GSH, and MDA levels nearly to normal mouse levels.

### 3.3. Effects of LP-HFY11 on the Levels of Serum Cytokines IL-2 and IL-10 in Mice

The findings presented in Table 6 indicate that the level of IL-2 in the blood was substantially higher, whereas that of IL-10 was significantly lower in the normal group (*p* < 0.05) compared with the other groups. The serum levels of IL-2 and IL-10 in the gatifloxacin and LP-HFY11H groups were closest to those in the normal group. The IL-2 levels in the LP-HFY11H groups were higher compared to the model group, whereas the IL-10 levels were lower compared with those in the model group and the LP-HFY11L group. The aforementioned conclusions were drawn by comparing the data of each group.

### 3.4. Pathological Observation of Mouse Colon Tissue

**Figure 1:**
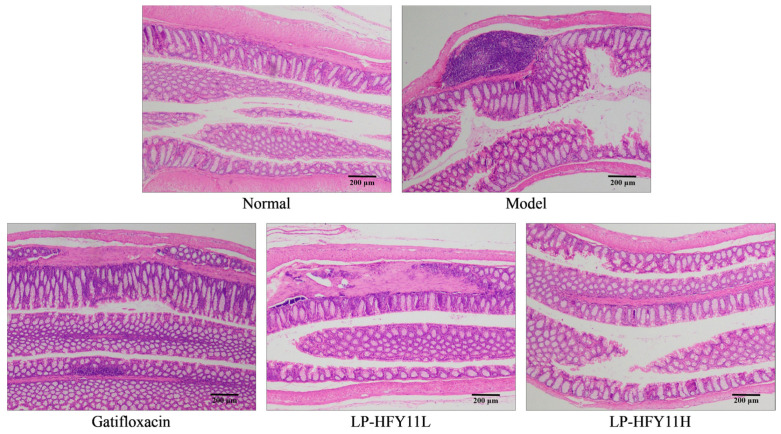
Effects of LR-AFY06 on the histopathology of mouse colon tissues (40×).

The H&E staining results showed that the mucosal epithelial cells of the colon tissue of the normal-group mice were intact, with normal crypts, cleanly distributed glands, and no ulcers (Figure 1). Numerous necrotic lesions and crypt abscesses were the result of a high number of inflammatory cells infiltrating into the colon of the model-group mice. In the gatifloxacin group, less inflammatory cell infiltration and less structural damage to the crypts were observed. The crypt structure was mostly intact, although the LP-HFY11 group had modest inflammatory infiltration. High concentrations of LP-KSFY01 considerably reduced pathological damage to the colon tissue compared with low concentrations of LP-HFY11.

### 3.5. Effects of LP-HFY11 on the mRNA Expression of nNOS, eNOS, and iNOS in Mouse Colon Tissue

**Figure 2:**
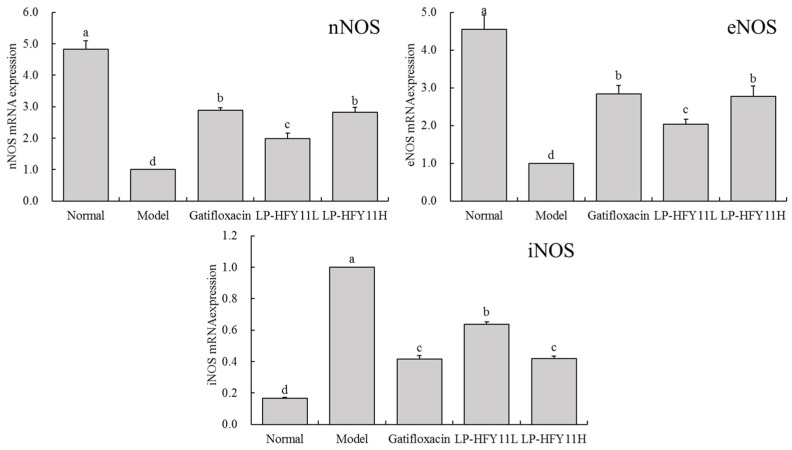
mRNA expression of nNOS, eNOS, and iNOS in mouse colon tissue. ^a–d^ The different letters denote statistically significant differences (*p* < 0.05) in the data’s mean values between the experimental groups (*n* = 10).

Figure 2 shows that the nNOS and eNOS mRNA expression levels were the most significant, whereas the iNOS expression level was the lowest, in the colon tissue of the normal-group mice. On the contrary, the iNOS expression level was the highest, whereas the nNOS and eNOS expression levels were the lowest in the model-group mice. The findings of the quantitative analysis demonstrate that gatifloxacin and LP-HFY11H only marginally reduced the expression levels of nNOS and eNOS in the colon of mice compared with those in the normal group; however, the iNOS expression level was only marginally higher.

### 3.6. Effects of LP-HFY11 on the mRNA Expression of c-Kit and SCF in Mouse Colon Tissue

**Figure 3:**
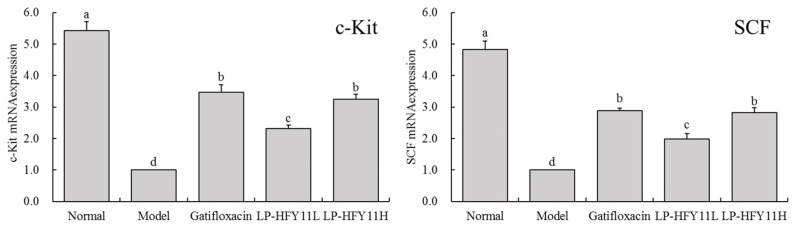
mRNA expression of c-Kit and SCF in mouse colon tissue. ^a–d^ The different letters denote statistically significant differences (*p* < 0.05) in the data’s mean values between the experimental groups (*n* = 10).

Based on the data shown in Figure 3, it was inferred that the c-Kit and SCF mRNA expression levels were considerably higher (*p* < 0.05) in the group treated with gatifloxacin and LP-HFY11 compared with the model group. Furthermore, the colons of mice treated with gatifloxacin and LP-HFY11H had considerably higher expression levels of c-Kit and SCF compared with the colons of mice in the LP-HFY11L group.

### 3.7. Effects of LP-HFY11 on the mRNA Expression of IL-8 and CXCR2 in Mouse Colon Tissue

**Figure 4:**
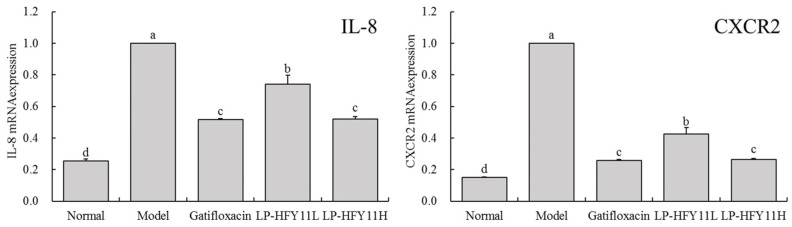
mRNA expression of IL-8 and CXCR2 in mouse colon tissue. ^a–d^ The different letters denote statistically significant differences (*p* < 0.05) in the data’s mean values between the experimental groups (*n* = 10).

The results in Figure 4 show that LP-HFY11H significantly reduced the expression levels of IL-8 and CXCR2 mRNA in the colon of mice (*p* < 0.05), but the levels were marginally higher than those in the gatifloxacin group. This was in contrast to the levels in the model and LP-HFY11L groups.

### 3.8. Effects of LP-HFY11 on the mRNA Expression of Microorganisms in Mouse Intestinal Contents 

**Figure 5:**
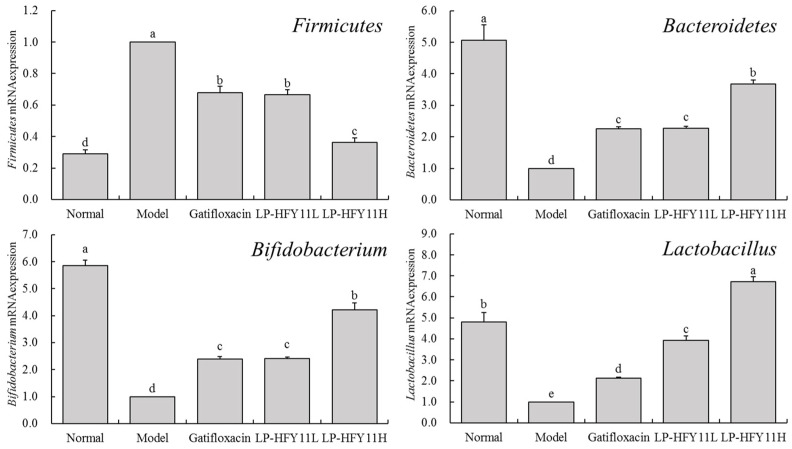
mRNA expression level of intestinal contents in mice. ^a–d^ The different letters denote statistically significant differences (*p* < 0.05) in the data’s mean values between the experimental groups (*n* = 10).

Figure 5 shows that *Firmicutes* mRNA expression was much lower in the intestinal contents of normal mice compared with mice in other groups (*p* < 0.05); however, *Bacteroidetes* and *Bifidobacterium* mRNA expression levels were significantly greater (*p* < 0.05). The *Firmicutes* mRNA expression level was the highest in the intestinal contents of the model-group mice, whereas the *Bacteroidetes*, *Lactobacillus*, and *Bifidobacterium* expression levels were the lowest. LP-HFY11 and gatifloxacin reduced the mRNA expression of *Firmicutes* in the intestinal contents of mice with thrombosis, while increasing the mRNA expression level of *Bacteroidetes* and *Bifidobacterium*. The mRNA expression level of *Lactobacillus* in the LP-HFY11H group was significantly higher than that in the normal group, and much higher than that in other groups (*p* < 0.05). The mRNA expression levels of *Bifidobacterium*, *Bacteroidetes,* and *Lactobacillus* in the intestinal contents of the LP-HFY11H group were significantly higher than those in the gatifloxacin group (*p* < 0.05). The expression ratio (F/B) of *Firmicutes*/*Bacteroidetes* was based on the normal state (ratio = 1). The model group reached 17.48, while the Gatifloxacin, LP-HFY11L, and LP-HFY11H groups were 5.28, 5.13, and 1.73, respectively.

## 4. Discussion

Colitis can cause weight loss, diarrhea, and other symptoms. DAI is a grading system for determining the severity of colitis based on body weight, stool parameters, and fecal blood [16]. According to the DAI index, we discovered that LP-HFY11 might alleviate the symptoms of oxazolone-induced colitis, with the impact becoming more significant when increasing the concentration of LP-HFY11. In addition, the colon length and colon weight-to-length ratio were used to determine the severity of colitis. Animals with colitis exhibited shorter colon lengths and a lower colon weight-to-length ratio than normal mice [17]. LP-HFY11 might potentially reduce the changes in colon length and weight induced by colitis.

MPO is an enzyme widely present in white blood cells, mainly in the granular body of neutrophils. In colitis, inflammatory responses lead to the aggregation and activation of neutrophils, resulting in the release of a large amount of MPO. MPO plays an essential role in colitis and is closely related to the development of inflammation and tissue damage [18]. It produces reactive oxygen species through oxidative reactions, such as hypochlorite ions and free radicals, which have a strong oxidative capacity and can cause the lipid peroxidation of cell membranes, leading to cell damage and further aggravating the inflammatory process [19]. In addition, MPO also produces nitric oxide (NO) from nitrous oxide (NO) and is involved in the regulation of inflammatory signaling pathways [20]. In colitis, excessive MPO production can damage the intestinal mucosal barrier, leading to cell damage and the further development of inflammation. Studies have shown that the level of MPO in the tissues of patients with colitis is closely related to the severity of inflammation, and high levels of MPO activity are associated with the activity and deterioration of the disease [21]. GSH is an important antioxidant. In colitis, inflammatory reactions and oxidative stress can lead to a decrease in the GSH level. Many free radicals and oxidizing substances are produced in the inflammatory process of colitis, increasing the consumption of GSH within the cell and thereby reducing its antioxidant capacity [22]. MDA is a product of lipid peroxidation and can cause lipid peroxidation in the cell membrane. In colitis, the production of MDA increases due to the increase in the inflammatory reaction and oxidative stress, aggravating the oxidative damage of the cell membrane and thus resulting in an impaired cell function. MDA, as a product of oxidative stress, can cause a further aggravation of inflammatory reactions [23]. It can activate inflammatory cells, such as neutrophils and macrophages, and release inflammatory mediators and cytokines, thus causing the expansion and continuity of inflammatory reactions. In addition, MDA is also associated with tissue damage caused by inflammation, leading to damage to epithelial cells and the destruction of the mucosal barrier function [24]. The regulatory effect of LP-HFY11 on the levels of MPO, NO, GSH, and MDA in mouse colon tissue may also be an essential mechanism underlying its alleviating effect on mouse colitis.

Colitis is intestinal tissue inflammation, and IL-2 is a cytokine promoting the inflammatory response. In colitis, immune cells produce a considerable amount of IL-2 in response to inflammation, exacerbating the inflammatory response. IL-2 is a key T-cell proliferation factor that promotes the proliferation and differentiation of CD^4+^ helper T cells and regulates the activity of various T-cell subsets. Colitis is characterized by an imbalance and aberrant function of T cells, as well as a compromised mucosal barrier function, allowing bacteria and toxins to infiltrate and induce an inflammatory response [25]. IL-2 regulates the immunological response of T cells and is vital for maintaining the immune balance and controlling intestinal inflammation. It is directly associated with the integrity of the intestinal mucosal barrier, emphasizing its importance in preventing the conditions that lead to colitis. IL-2 helps regulate the integrity of the mucosal barrier and maintain a proper intestinal function [26]. In particular, IL-10 is essential for regulating intestinal inflammation and other immune responses. As macrophages are the primary target cells for the anti-inflammatory activity of IL-10, this impact is most noticeable in these cells. Second, IL-10 regulates the metabolic activities of macrophages to alleviate inflammation somewhat. It causes macrophages to switch from glycolysis to oxidative phosphorylation metabolism, which is linked to an anti-inflammatory phenotype. Third, mTOR inhibition is significantly aided by IL-10 [27]. The maintenance of macrophage mitochondrial health and the promotion of phagocytosis depend on this suppression of mTOR. Finally, impaired IL-10 signaling causes the accumulation of damaged mitochondria in macrophages, which adds to the dysregulated inflammatory response seen in patients with inflammatory bowel disease [28]. Hence, it is presumed that LP-HFY11 can regulate the validation cytokines in serum, thereby intervening in the development of colitis.

nNOS mainly exists in nerve tissue, and its function is to synthesize nitric oxide (NO). It regulates neural signal transmission by releasing NO. In colitis, nNOS is involved in regulating intestinal motility, blood flow, and the protection of intestinal mucosa [29]. eNOS is mainly distributed in endothelial cells. It is responsible for synthesizing nitric oxide and plays a role in regulating vasodilation, as well as anti-inflammatory and anticoagulant effects. In colitis, eNOS is involved in regulating the intestinal blood flow, protecting the mucosal barrier, and inhibiting the inflammatory response [30]. iNOS is mainly induced and expressed during the inflammatory process, and its synthesis ability is high, producing a large amount of NO. In colitis, the expression of iNOS is usually associated with an inflammatory response. It can participate in the bactericidal effect of inflammatory cells, regulate the immune response, and regulate inflammatory signaling pathways by releasing NO [31]. nNOS, eNOS, and iNOS play important roles in colitis. They participate in regulating physiological and pathological processes, such as intestinal motility, blood flow, the mucosal barrier, and the inflammatory response, through NO synthesis [32].

c-Kit is mainly expressed in intestinal interstitial cells (Cajal cells) and mucosal epithelial cells. It participates in regulating intestinal motility, maintaining mucosal barrier integrity, and regulating immune responses by binding to its ligand SCF [33]. SCF is mainly produced by intestinal epithelial and stromal cells. It can bind to c-Kit receptors, promote the proliferation and differentiation of Cajal cells, regulate intestinal smooth muscle contraction and movement, and participate in intestinal repair and mucosal barrier protection [34]. In colitis, the functions of c-Kit and SCF may be disrupted. The inflammatory response leads to the abnormal activation of the c-Kit signaling pathway and the abnormal expression of SCF. This may affect the normal motility of the intestine, the integrity of the mucosal barrier, and the regulation of the immune response, further exacerbating the inflammatory process [35].

IL-8 is a chemotactic factor mainly produced by various cells, such as inflammatory, epithelial, and endothelial cells. Its primary purpose is to attract and activate white blood cells, particularly neutrophils, thus promoting their migration to the site of inflammation. Excessive IL-8 production can cause inflammation to persist, thus aggravating colitis [36]. CXCR2 is an IL-8 receptor mainly expressed on the surface of white blood cells. When IL-8 binds to CXCR2, it activates the CXCR2 signaling pathway, triggering various inflammatory responses in cells [37]. In colitis, the activation of CXCR2 can enhance neutrophil chemotaxis and activation, exacerbating the inflammatory response and causing tissue damage. The aberrant expression of IL-8 and CXCR2 may result in the increased aggregation and activation of inflammatory cells, prolonging and exacerbating the inflammatory response. As a result, blocking the IL-8 and CXCR2 signaling pathways may be a useful approach for treating colitis because it can reduce inflammation and tissue damage [38]. The mRNA regulatory effect of LP-HFY11 on colon tissue can effectively intervene in the development of colitis in mice, thereby promoting functional recovery.

Dysfunction of gut microbiota is an essential factor leading to inflammation of the colon. A vast majority of bacteria in the human gut are composed of *Firmicutes* and *Bacteroidetes*, and a higher proportion of *Firmicutes* than *Bacteroidetes* can cause an increase in the levels of inflammatory factors in the blood, increasing the possibility of intestinal tissue damage [39]. The gut *Firmicutes*/*Bacteroidetes* ratio (F/B) is widely believed to have an important impact on maintaining normal intestinal stability. Experimental colitis has shown that the F/B ratio in the gut increases after the induction of colitis, which can be used as an indicator to evaluate the stability of gut microbiota [40]. *Lactobacillus* and *Bifidobacteria* exert various bioactive effects, especially in protecting the normal metabolic state of the body, avoiding tissue damage caused by inflammation and other abnormalities and thus protecting the intestine [41]. In addition, a healthy gut microbiota can improve the body’s resistance and tissue-repair ability by regulating various mechanisms, such as the immune function, and intervene in colitis [42]. This study also confirmed that LP-HFY11 promoted the production of *Bacteroidetes* and *Bifidobacteria* in the mouse intestine, while supplementing with *Lactobacillus* and reducing the expression of *Firmicutes* and the F/B expression ratio, thereby inhibiting inflammation by regulating the intestinal microbiota and exerting an inhibitory effect on colitis formation. In particular, the side effects of gatifloxacin as a drug include symptoms such as nausea, diarrhea, abdominal pain, constipation, and indigestion. As a lactic acid bacteria isolated from food, LP-HFY11 has no side effects and has better application prospects.

## 5. Conclusions

This preliminary study investigated the inhibitory effect of LP-HFY11 on oxazolone-induced colitis. Animal experiments have shown that LP-HFY11 effectively reduces symptoms in mice with colitis and regulates factors related to inflammation and immunity in colon tissue and serum. Meanwhile, this study further demonstrated the ability of LP-HFY11 to control the gut microbiota composition of mice, improving it in the process. This preserves bodily health, lowers inflammation, and effectively stops the progression of colitis. This indicates that LP-HFY11 has the potential to be developed into a probiotic and can successfully intervene in oxazolone-induced colitis.

## Figures and Tables

**Table 1:** DAI score benchmark.

Percentage of Body Weight Loss (%)	Stool Consistency	Presence of Blood in Stool	Score
No decrease	Normal	Occult blood (−)	0
1–5			1
5–10	Semi-loose stool	Occult blood (+)	2
10–15			3
>15	Loose stools	Naked-eye bloody stool	4

**Table 2:** Primer sequence for the reverse transcription–polymerase chain reaction.

Gene	Primer Sequence
nNOS	F: 5′-TGCTCTGTGAATCCACCACC-3′
	R: 5′-GAGGAAGCGAAAGCACTCCAG-3′
eNOS	F: 5′-TCAGCCATCACAGTGTTCCC-3′
	R: 5′-ATAGCCCGCATAGCGTATCAG-3′
iNOS	F: 5′-GGAGTGACGGCAAACATGACT-3′
	R: 5′-TCGATGCACAACTGGGTGAAC-3′
c-Kit	F: 5′-AGACCGAACGCAACTT-3′
	R: 5′-GGTGCCATCCACTTCA-3′
SCF	F: 5′-AAACTGGTGGCGAATC-3′
	R: 5′-CACGGGTAGCAAGAAC-3′
IL-8	F: 5′-GGCCTTGTAGACACCTTGGT-3′
	R: 5′-GAACAA AGGCAAGGCTAA-3′
CXCR2	F: 5′-ATGCCCTCTATTCTGCCAGAT-3′
	R: 5′-GTGCTCCGGTTGTATAAGATGAC-3′
GAPDH	F: 5′-AATGGATTTGGACGCATTGGT-3′
	R: 5′-TTTGCACTGGTACGTGTTGAT-3′
*Firmicutes*	F: 5′-GCGTGAGTGAAGAAGT-3′
	R: 5′-CTACGCTCCCTTTACAC-3′
*Bacteroidetes*	F: 5′-ACGCTAGCTACAGGCTTAACA-3′
	R: 5′-ACGCTACTTGGCTGGTTCA-3′
*Lactobacillus*	F: 5′-CACCGCTACACATGGAG-3′
	R: 5′-AGCAGTAGGGAATCTTCCA-3′
*Bifidobacterium*	F: 5′-TCGCGTCYGGTGTGAAAG-3′
	R: 5′-CCACATCCAGCRTCCAC-3′
Total bacteria	F: 5′-ACTCCTACGGGAGGCAGCAGT-3′
	R: 5′-ATTACCGCGGCTGCTGGC-3′

**Table 3:** DAI value for each group of mice (*n* = 10).

Group	On the 1st Day after Enema	On the 3rd Day after Enema	On the 7th Day after Enema
Normal	0.00 ± 0.00 ^c^	0.00 ± 0.00 ^c^	0.00 ± 0.00 ^c^
Model	1.83 ± 0.18 ^a^	2.71 ± 0.21 ^a^	3.96 ± 0.12 ^a^
Gatifloxacin	0.58 ± 0.30 ^ab^	1.50 ± 0.25 ^ab^	1.88 ± 0.25 ^ab^
LP-HFY11L	1.04 ± 0.28 ^b^	1.95 ± 0.28 ^b^	2.42 ± 0.30 ^b^
LP-HFY11H	0.67 ± 0.25 ^ab^	1.63 ± 0.21 ^ab^	1.96 ± 0.21 ^ab^

^a–c^ The different letters denote statistically significant differences (*p* < 0.05) in the data’s mean values between the experimental groups.

**Table 4:** Colon length and weight in every group (*n* = 10).

Group	Colon Length (cm)	Colon Weight/Length (mg/cm)
Normal	9.85 ± 0.33 ^a^	39.15 ± 3.5 ^a^
Model	5.70 ± 0.27 ^d^	23.53 ± 5.10 ^c^
Gatifloxacin	8.24 ± 0.25 ^b^	35.07 ± 3.43 ^ab^
LP-HFY11L	6.65 ± 0.33 ^c^	30.46 ± 6.48 ^b^
LP-HFY11H	8.15 ± 0.44 ^b^	32.95 ± 2.53 ^ab^

^a–d^ The different letters denote statistically significant differences (*p* < 0.05) in the data’s mean values between the experimental groups.

**Table 5:** MPO, NO, GSH, and MDA levels in the colon tissue of each mouse group (*n* = 10).

Group	MPO (mU/mg)	NO (μmol/gprot)	GSH (µmol/mg)	MDA (nmol/mg)
Normal	28.33 ± 4.13 ^d^	0.58 ± 0.07 ^c^	21.79 ± 2.96 ^a^	0.52 ± 0.03 ^d^
Model	238.39 ± 14.95 ^a^	5.28 ± 1.00 ^a^	3.49 ± 1.02 ^d^	1.79 ± 0.13 ^a^
Gatifloxacin	121.99 ± 5.58 ^c^	2.62 ± 0.52 ^b^	14.58 ± 2.98 ^b^	0.94 ± 0.09 ^c^
LP-HFY11L	179.51 ± 7.00 ^b^	4.36 ± 0.96 ^a^	9.19 ± 1.94 ^c^	1.47 ± 0.11 ^b^
LP-HFY11H	125.43 ± 8.26 ^c^	2.67 ± 0.41 ^b^	13.73 ± 1.67 ^b^	0.97 ± 0.06 ^c^

^a–d^ The different letters denote statistically significant differences (*p* < 0.05) in the data’s mean values between the experimental groups.

**Table 6:** IL-2 and IL-10 serum cytokine levels in each group (*n* = 10).

Group	IL-2 (pg/mL)	IL-10 (pg/mL)
Normal	192.99 ± 4.71 ^a^	251.91 ± 10.67 ^d^
Model	16.03 ± 7.10 ^d^	619.34 ± 17.89 ^a^
Gatifloxacin	141.75 ± 8.34 ^b^	366.29 ± 14.94 ^c^
LP-HFY11L	52.63 ± 7.15 ^c^	548.01 ± 17.52 ^b^
LP-HFY11H	138.49 ± 6.79 ^b^	375.07 ± 17.78 ^c^

^a–d^ The different letters denote statistically significant differences (*p* < 0.05) in the data’s mean values between the experimental groups.

## Data Availability

The original contributions presented in the study are included in the article, further inquiries can be directed to the corresponding author.

## References

[1] Zhao X., Qian Y., Li G., Yi R., Park K.Y., Song J.L. (2019). Lactobacillus plantarum YS2 (yak yogurt Lactobacillus) exhibited an activity to attenuate activated carbon-induced constipation in male Kunming mice. J. Dairy Sci..

[2] Luo F., Feng S., Sun Q., Xiang W., Zhao J., Zhang J., Yang Z. (2011). Screening for bacteriocin-producing lactic acid bacteria from kurut, a traditional naturally-fermented yak milk from Qinghai–Tibet plateau. Food Cont..

[3] van der Logt E.M.J., Blokzijl T., van der Meer R., Faber K.N., Dijkstra G. (2013). Westernized high-fat diet accelerates weight loss in dextran sulfate sodium-induced colitis in mice, which is further aggravated by supplementation of heme. J. Nutr. Biochem..

[4] Xu P., Luo S., Song J., Dai Z., Li D., Wu C. (2022). Effect of sodium alginate-based hydrogel loaded with lutein on gut microbiota and inflammatory response in DSS-induced colitis mice. Food Sci. Hum. Well..

[5] Floudas A., Neto N., Orr C., Canavan M., Gallagher P., Hurson C., Monaghan M.G., Nagpar S., Mullan R.H., Veale D.J. (2022). Loss of balance between protective and pro-inflammatory synovial tissue T-cell polyfunctionality predates clinical onset of rheumatoid arthritis. Ann. Rheum. Dis..

[6] Hunter M.M., Wang A., McKay D.M. (2007). Helminth infection enhances disease in a murine TH2 model of colitis. Gastroenterology.

[7] Rong L., Liu W., Ding W., Jiang Y., Zhong L., Sun D. (2007). Modulation of dendritic cell phenotype and its function by probiotics in experimental colitis in rats. Chin. J. Gastroenterol..

[8] Dhillon P., Singh K. (2020). Therapeutic applications of probiotics in ulcerative colitis: An updated review. Pharma Nutr..

[9] Adolfsson O., Meydani S.N., Russell R.M. (2004). Yogurt and gut function. Am. J. Clin. Nutr..

[10] Wildt S., Munck L.K., Vinter-Jensen L., Hanse B.F., Nordgaard-Lassen I., Christensen S., Avnstroem S., Rasmussen S.N., Rumessen J.J. (2006). Probiotic treatment of collagenous colitis: A randomized, double-blind, placebo-controlled trial with *Lactobacillus acidophilus* and *Bifidobacterium animalis* subsp. Lactis. Inflamm. Bowel. Dis..

[11] Daniel C., Poiret S., Goudercourt D., Dennin V., Leyer G., Pot B. (2006). Selecting lactic acid bacteria for their safety and functionality by use of a mouse colitis model. Appl. Environ. Microbiol..

[12] Fitzpatrick L.R. (2013). Probiotics for the treatment of *Clostridium difficile* associated disease. World J. Gastrointest. Pathophysiol..

[13] Watanabe T., Yamamoto T., Yoshida M., Fujiwara K., Kageyama-Yahara N., Kuramoto H., Shimada Y., Kadowaki M. (2010). The traditional herbal medicine saireito exerts its inhibitory effect on murine oxazolone-induced colitis via the induction of Th1-polarized immune responses in the mucosal immune system of the colon. Int. Arch. Allergy. Immunol..

[14] Zhao X., Yi R., Zhou X., Mu J., Long X., Pan Y., Song J.L., Park K.Y. (2019). Preventive effect of Lactobacillus plantarum KSFY02 isolated from naturally fermented yogurt from Xinjiang, China, on d-galactose–induced oxidative aging in mice. J. Dairy Sci..

[15] Hu T., Chen R., Qian Y., Ye K., Long X., Park K.Y., Zhao X. (2022). Antioxidant effect of Lactobacillus fermentum HFY02-fermented soy milk on D-galactose-induced aging mouse model. Food Sci. Hum. Well..

[16] Du C., Li Z., Zhang J., Yin N., Tang L., Li J., Sun J., Yu X., Chen W., Xiao H. (2023). The protective effect of carnosic acid on dextran sulfate sodium-induced colitis based on metabolomics and gut microbiota analysis. Food Sci. Hum. Well..

[17] Azuma K., Osaki T., Tsuka T., Imagawa T., Okamoto Y., Minami S. (2014). Effects of fish scale collagen peptide on an experimental ulcerative colitis mouse model. Pharma Nutr..

[18] Chen M., Yao H., Tan G., Huang W., Wu Q., Nie S. (2022). Impact of Bifidobacterium longum NSP001 on DSS-induced colitis in conventional and humanised mice. Food Sci. Hum. Well..

[19] Chen L., You Q., Hu L., Gao J., Meng Q., Liu W., Wu X., Xu Q. (2018). The antioxidant procyanidin reduces reactive oxygen species signaling in macrophages and ameliorates experimental colitis in mice. Front. Immunol..

[20] Abd Elmaksoud H.A., Motawea M.H., Desoky A.A., Elharrif M.G., Ibrahimi A. (2021). Hydroxytyrosol alleviate intestinal inflammation, oxidative stress and apoptosis resulted in ulcerative colitis. Biomed. Pharmacother..

[21] Wang T., Wang P., Yin L., Wang X., Shan Y., Yi Y., Zhou Y., Liu B., Wang X., Lü X. (2023). Dietary Lactiplantibacillus plantarum KX041 attenuates colitis-associated tumorigenesis and modulates gut microbiota. Food Sci. Hum. Well..

[22] Özbeyli D., Berberoglu A.C., Özen A., Erkan O., Başar Y., Şen T., Akakın D., Yüksel M., Çakır Ö.K. (2017). Protective effect of alpha-lipoic acid, aerobic or resistance exercise from colitis in second hand smoke exposed young rats. Clin. Exp. Pharmacol. Physiol..

[23] Xie J., Liu L., Li H., Che H., Xie W. (2022). Ink melanin from *Sepiapharaonis ameliorates* colitis in mice via reducing oxidative stress, andprotecting the intestinal mucosal barrier. Food. Res. Int..

[24] Wen Y., Xiao H., Liu Y., Yang Y., Wang Y., Xu S., Huang S., Hou S., Liang J. (2021). Polysaccharides from Dendrobium officinale ameliorate colitis-induced lung injury via inhibiting inflammation and oxidative stress. Chem. Biol. Interact..

[25] Yang W., Yao Y., Yang Y.Q., Lu F.T., Li L., Wang Y.H., Nakajima T., Tsuneyama K., Ridgway W.M., Gershwin M.E. (2014). Differential modulation by IL-17A of Cholangitis versus Colitis in IL-2Rα deleted mice. PLoS ONE.

[26] Yamanaka K., Saito J., Nakata W., Sato M., Abe T., Mori N., Sekii K., Yoshioka T., Itatani H. (2009). Case of drug-induced colitis like ulcerative colitis during IL-2 therapy for multiple bone metastasis after operation of kidney cancer. Jpn. J. Urol..

[27] Wu Q., Xie S., Zhu Y., Chen J., Tian J., Xiong S., Wu C., Ye Y., Peng Y. (2021). Wogonin strengthens the therapeutic effects of mesenchymal stem cells in DSS-induced colitis via promoting IL-10 production. Oxid. Med. Cell. Longev..

[28] Hu S., Chen M., Wang Y., Wang Z., Pei Y., Fan R., Liu X., Wang L., Zhou J., Zheng S. (2016). mTOR inhibition attenuates dextran sulfate sodium-induced colitis by suppressing T cell proliferation and balancing TH1/TH17/Treg profile. PLoS ONE.

[29] Kono T., Chisato N., Ebisawa Y., Asama T., Sugawara M., Ayabe T., Kohgo Y., Kasai S., Yoneda M., Takahashi T. (2004). Impaired nitric oxide production of the myenteric plexus in colitis detected by a new bioimaging system. J. Surg. Res..

[30] Okaniwa N., Sasaki M., Mizushima T., Ogasawara N., Funaki Y., Joh T., Kasugai K. (2015). eNOS plays an important role in the regulation of colonic inflammation: A novel therapeutic target and a predictive marker for the prognosis of ulcerative colitis. Free Radic. Res..

[31] Mazzon E., Cuzzocrea S. (2003). Role of iNOS in hepatocyte tight junction alteration in mouse model of experimental colitis. Cell Mol. Biol..

[32] Qian Y., Yi R., Sun P., Li G., Zhao X. (2017). Lactobacillus plantarum YS2 reduces oxazolone-induced colitis in BALB/c mice. Biomed. Res..

[33] Kim H.J., Kim N., Kim Y.S., Nam R.H., Lee S.M., Park J.H., Choi D., Hwang Y.J., Lee J., Lee H.S. (2017). Changes in the interstitial cells of Cajal and neuronal nitric oxide synthase positive neuronal cells with aging in the esophagus of F344 rats. PLoS ONE.

[34] Chen Y., Xu J., Liu S., Hou X. (2013). Electroacupuncture at ST36 increases contraction of the gastric antrum and improves the SCF/c-kit pathway in diabetic rats. Am. J. Chin. Med..

[35] Long X., Pan Y., Zhao X. (2018). Prophylactic effect of Kudingcha polyphenols on oxazolone induced colitis through its antioxidant capacities. Food Sci. Hum. Well..

[36] Li J., Moran T., Swanson E., Julian C., Harris J., Bonen D.K., Hedl M., Nicolae D.L., Abraham C., Cho J.H. (2004). Regulation of IL-8 and IL-1beta expression in Crohn’s disease associated NOD2/CARD15 mutations. Hum. Mol. Genet..

[37] Katoh H., Wang D., Daikoku T., Sun H., Dey S.K., Dubois R.N. (2013). CXCR2-expressing myeloid-derived suppressor cells are essential to promote colitis-associated tumorigenesis. Cancer Cell.

[38] Marchelletta R.R., Gareau M.G., Okamoto S., Guiney D.G., Barrett K.E., Fierer J. (2014). Salmonella-induced diarrhea occurs in the absence of IL-8 receptor (CXCR2)-dependent neutrophilic inflammation. J. Infect. Dis..

[39] Parnell J.A., Reimer R.A. (2012). Prebiotic fibres dose-dependently increase satiety hormones and alter Bacteroidetes and Firmicutes in lean and obese JCR:LA-cp rats. Br. J. Nutr..

[40] Yu Z.T., Li D.G., Sun H.X. (2023). Herba Origani alleviated DSS-induced ulcerative colitis in mice through remolding gut microbiota to regulate bile acid and short-chain fatty acid metabolisms. Biomed. Pharmacother..

[41] Zhou B., Jin G., Pang X., Mo Q., Bao J., Liu T., Wu J., Xie R., Liu X., Liu J. (2022). *Lactobacillus rhamnosus* GG colonization in early life regulates gut-brain axis and relieves anxiety-like behavior in adulthood. Pharmacol. Res..

[42] Garrett W.S., Gallini C.A., Yatsunenko T., Michaud M., DuBois A., Delaney M.L., Punit S., Karlsson M., Bry L., Glickman J.N. (2010). Enterobacteriaceae act in concert with the gut microbiota to induce spontaneous and maternally transmitted colitis. Cell Host Microbe.

